# The genomic landscape of estrogen receptor α binding sites in mouse mammary gland

**DOI:** 10.1371/journal.pone.0220311

**Published:** 2019-08-13

**Authors:** Murugesan Palaniappan, Loc Nguyen, Sandra L. Grimm, Yuanxin Xi, Zheng Xia, Wei Li, Cristian Coarfa

**Affiliations:** 1 Department of Molecular and Cellular Biology, Baylor College of Medicine, Houston, United States of America; 2 Division of Biostatistics, Dan L. Duncan Cancer Center, Baylor College of Medicine, Houston, United States of America; 3 Dan L. Duncan Cancer Center, Baylor College of Medicine, Houston, United States of America; 4 Advanced Technology Core, Baylor College of Medicine, Houston, United States of America; Universita degli Studi della Campania Luigi Vanvitelli, ITALY

## Abstract

Estrogen receptor α (ERα) is the major driving transcription factor in the mammary gland development as well as breast cancer initiation and progression. However, the genomic landscape of ERα binding sites in the normal mouse mammary gland has not been completely elucidated. Here, we mapped genome-wide ERα binding events by chromatin immunoprecipitation followed by high-throughput sequencing (ChIP-seq) in the mouse mammary gland in response to estradiol. We identified 6237 high confidence ERα binding sites in two biological replicates and showed that many of these were located at distal enhancer regions. Furthermore, we discovered 3686 unique genes in the mouse genome that recruit ER in response to estradiol. Interrogation of ER-DNA binding sites in ER-positive luminal epithelial cells showed that the ERE, PAX2, SF1, and AP1 motifs were highly enriched at distal enhancer regions. In addition, comprehensive transcriptome analysis by RNA-seq revealed that 493 genes are differentially regulated by acute treatment with estradiol in the mouse mammary gland *in vivo*. Through integration of RNA-seq and ERα ChIP-seq data, we uncovered a novel ERα targetome in mouse mammary epithelial cells. Taken together, our study has identified the genomic landscape of ERα binding events in mouse mammary epithelial cells. Furthermore, our study also highlights the cis-regulatory elements and cofactors that are involved in estrogen signaling and may contribute to ductal elongation in the normal mouse mammary gland.

## Introduction

Estrogen is a key hormone for mammary epithelial proliferation during the mammary gland development, as well as during breast cancer initiation and progression [1–4]. The genomic action of estrogen is mediated through two members of the nuclear receptor family, estrogen receptor α (ERα) and ERβ [5]. Of these two receptors, ERα plays a dominant role in mammary gland development, function, and tumorigenesis [6–9]. The classical pathway of estrogen action involves estrogen binding to the estrogen receptor, resulting in receptor dimerization and ligand-receptor complex entering into the nucleus where it then binds directly to the genomic DNA containing estrogen response elements (EREs) of the target genes. Various coregulators are then recruited to the gene promoter and/or distal enhancer region to modulate ER-mediated gene transcription [10, 11]. In the non-classical pathway, ligand-receptor complexes bind indirectly to genomic regions by tethering to other transcription factors, including the activator protein 1 (AP-1) families of transcription factors (TFs), members of the specificity protein 1 (SP1), and nuclear factor kappa-light-chain-enhancer of activated B cells (NF-κB), which lead to recruitment of chromatin-modifying coregulator proteins and allow activation or repression of ER target genes to drive cell proliferation [12–14].

In the mammary gland, ERα is the major receptor operating during ductal morphogenesis, as determined by the analysis of knockout phenotypes in mice [15]. Studies have shown that deletion of ERα inhibits development of the rudimentary ductal structure, and signaling from estradiol through ERα during puberty is required for mammary epithelial proliferation, ductal elongation, bifurcation, and invasion throughout the mammary fat pad [8, 16, 17]. Further, it has been shown that only a subset of luminal epithelial cells express ERα and estrogen promotes mammary epithelial cell proliferation by inducing amphiregulin (*Areg*), an epidermal growth factor (EGF) receptor ligand produced in ERα positive cells. *Areg* acts through a paracrine mechanism on neighboring ER-negative mammary epithelial and stromal cells to mediate estrogen-induced cell proliferation that drives ductal elongation [18, 19]. The processes of normal postnatal mammary gland development display many of the properties associated with breast cancer progression such as proliferation, invasion, angiogenesis, and resistance to apoptosis [20].

Several studies have focused on studying genome-wide ER binding sites in ER-positive breast cancer cell lines [21–26]. This approach has helped to identify the cis-regulatory elements and cofactors that are involved in mediating ER binding and ER target gene transcription in breast cancer cells. These studies have shown that the vast majority of ER binding sites are located at distal enhancer regions [21–23]. Furthermore, Brown and colleagues discovered that ER binding regions were enriched for the FOXA1 binding motif. Subsequently, FOXA1 was then identified as a pioneer factor for ER-chromatin interactions in ER-positive breast cancer cells [27, 28]. Genome-wide mapping of FOXA1 binding demonstrated that more than 50% of FOXA1 binding sites overlapped with ER binding events in ER-positive breast cancer cells [29]. In addition to FOXA1, studies have implicated GATA3 and PBX1 as pioneer factors for ER-chromatin interactions, since depletion of these transcription factors leads to reduction of ER binding events and transcriptional activity in response to estrogen [30, 31]. Furthermore, it has been shown that GATA3 acts upstream of FOXA1 in the ER binding [31].

Recently, Carroll and colleagues also mapped ER binding events in primary breast cancer samples from patients with different clinical outcomes [32]. The authors found that a subset of ER binding sites is maintained in good outcome, poor outcome, and metastatic breast tumors. Differential binding analysis revealed that ER binding events can discriminate between good and poor outcome tumors. Furthermore, motif analysis revealed that ERE motif is only enriched in good outcome ER-bound genomic regions, indicating that FOXA1 is not involved in these ER binding regions. On the other hand, in poor outcome tumors, ER binding events were associated with ERE and FOXA1 motifs. Nonetheless, it should be noted that genome-wide ER binding studies have been limited to ER-positive breast cancer cell lines and primary breast cancer samples, not the normal mammary gland. Although one recent study has shown ER binding sites in the developing mouse mammary gland, this study focused only on control mammary glands which have not received any hormonal treatment [33].

Given the potential involvement of ERα in mammary gland development, as well as in breast cancer initiation and progression, we investigated for the first time, genome-wide ERα binding events by chromatin immunoprecipitation followed by high-throughput sequencing in mouse mammary gland under *in vivo* conditions of acute treatment with estradiol. Furthermore, we also used genome-wide transcriptome profiling (RNA-seq) to uncover the global transcriptome response to estradiol. This combined system approaches allowed us to identify the ER targetome in the mammary gland and this ER targetome consists of a subset of ER-regulated target genes whose acute regulation by estradiol is associated with direct binding to ER in luminal epithelial cells. Overall, our study provides a unique resource for the mechanisms underlying estrogen regulated gene expression in the mouse mammary gland.

## Materials and methods

### Mice

Animal experiments were approved by the Institutional Animal Care and Use Committee (IACUC) at Baylor College of Medicine (Houston, TX). BALB/cJ mice were purchased from The Jackson Laboratory (Bar Harbor, ME). All mice were housed in the animal facility at Baylor College of Medicine and maintained in a conventional mouse facility with room temperature set at 22°C with food and water provided *ad libitum*. The animal facility is accredited by the American Association of Laboratory Animal Care.

#### Hormone treatments

At 6 weeks of age, mice were ovariectomized and rested for 10 days and then mice were injected subcutaneously with sesame oil (50μl) or 17β-estradiol (100ng) for 2 hours. Sesame oil and 17β-estradiol were purchased from Sigma. After 2 hours, mice were sacrificed and both inguinal mammary glands (#4 mammary glands) were harvested from each mouse. The lymph node was removed from the # 4 mammary glands and used for RNA-seq and ChIP-seq analysis.

#### Total RNA isolation

For RNA-seq and quantitative Real-Time PCR (qPCR), total mammary gland RNA was isolated from #4 glands using the RNeasy Lipid Tissue Midi Kit according to the manufacturer’s instructions (QIAGEN, Inc., Valencia, CA). To avoid potential contamination from muscle, only inguinal (rather than thoracic) glands were used in this study. Physical integrity of the RNA was assessed using the Agilent 2100 Bioanalyzer (Agilent Technologies, Inc., Santa Clara, CA). RNA quantitation was performed using the Nanodrop ND1000 spectrophotometer (Nanodrop Technologies, Wilmington, DE). For qPCR and RNA-seq analysis, total RNA pooled from a set of four to five mice per treatment group was analyzed. To ensure statistical significance, three separate sets of mice (12–15 mice in total) per treatment group were used in qPCR experiment.

#### RNA-seq library preparation and sequencing

Total RNA samples with RNA integrity number (RIN) ≥8 were used for transcriptome sequencing. Total RNA (10ng) was used for amplified double-stranded cDNA using the Ovation RNA-Seq System (NuGEN, San Carlos, CA). Double-stranded DNA was sheared to 200-300bp using the Covaris S2 sonicator (Covaris, Woburn, MA) and ligated to Illumina paired-end adaptors using the Illumina TruSeq DNA library preparation kit according to the manufacturer’s instructions (Illumina, San Diego, CA). PCR amplification was performed to obtain the final cDNA library. Bioanalyzer 2100 (Agilent Technologies, Santa Clara, CA) analysis was used to verify fragment size after amplification, library size and concentration before clustering. A total of 10pM of the library was then used for paired-end sequencing on the HiSeq 2500 at the Genomic and RNA Profiling Core in Baylor College of Medicine.

#### Quantitative Real-Time PCR

RNA-seq was validated by qPCR. First, RNA was reverse transcribed using the High Capacity cDNA Reverse Transcription Kit (Applied Biosystems, Foster City, CA). TaqMan Universal PCR chemistry was implemented and tested on the ABI Prism PE7700 Sequence Detection System according to the manufacturer’s instructions (Applied Biosystems). Standard curves were generated using a serial dilution of Mouse Universal Reference Total RNA (Clontech, Mountain View, CA). All experiments were performed in triplicate using three independent cDNA sets per treatment and normalized to cyclophilin D (*Ppid*). TaqMan Primer probes were purchased from Applied Biosystems (Foster City, CA) and are as follows: *Greb1* (Mm00479269_m1), *Pgr* (Mm00435628_m1), *Fos* (Mm00487425_m1), *Areg* (Mm00437583_m1), *Ccnd1* (Mm00432359_m1), *Gata3* (Mm00484683_m1), *Foxa1* (Mm00484713_m1), *Cdh1* (Mm01247357_m1), *Krt8* (Mm04209403_g1), *Krt18* (Mm01601704_g1), *Krt7* (Mm00466676_m1), *Muc1* (Mm00449604_m1), *Krt4* (Mm01296260_m), *Csf1* (Mm00432686_m1), *Bmp8a* (Mm00432109_m), *Heyl* (Mm00516558_m1), *Errfi1* (Mm00505292_m1), *Six1* (Mm00808212_m1), *Gata6* (Mm00802636_m1), and *Ppid* (Mm00835365_g1).

#### ChIP-seq analysis

For chromatin immunoprecipitation coupled with parallel sequencing (ChIP-seq) analysis, #4 mammary glands were pooled from six mice per replicate and two biological replicates were used for ERα ChIP-seq. Mammary glands were cut into small pieces and fixed in 1% formaldehyde for 15 min and quenched with 0.125M glycine for 5 min at room temperature. Mammary gland chromatin was prepared using ChIP-IT High Sensitivity Kit (Active Motif, Carlsbad, CA) according to the manufacturer’s instructions. Briefly, the tissue pieces were homogenized using hand-held tissue homogenizer for 30 seconds and then spun down and washed twice with PBS. Chromatin was isolated from cell pellets by adding 5 ml of chromatin preparation buffer supplemented with Protease Inhibitor Cocktail and phenylmethanesulfonylfluoride (PMSF), followed by disruption of resuspended cell pellets with a Dounce homogenizer. Samples were pelleted by centrifugation and resuspended with ChIP buffer supplemented with protease inhibitor cocktail and PMSF. Lysates were sonicated and DNA was sheared to an average length of 150-500bp. Input DNA was prepared by treating aliquots with RNase, proteinase K, and heat to reverse crosslinks. The DNA was then purified by QIAquick PCR Purification Kit (QIAGEN, Inc., Valencia, CA) according to the manufacturer’s instructions. Sonication efficiency was verified by agarose gel electrophoresis.

An aliquot of chromatin (~ 60ug) was precleared with protein G agarose beads (Active Motif, Carlsbad, CA). ChIP reactions were performed using an antibody against ERα (sc-542, Santa Cruz Biotechnology, Santa Cruz, CA). After incubation at 4°C overnight, protein G agarose beads were used to isolate the immune complexes and were washed, eluted from beads with elution buffer, and then subjected to RNase and proteinase K treatment. Crosslinks were reversed by incubation overnight at 65°C and ChIP DNA was purified by DNA purification kit (Active Motif, Carlsbad, CA). ERα ChIP enrichment was verified by qPCR using positive (*Greb1*) and negative (*Untr6*) targets. The resulting signals were normalized to input DNA.

#### ChIP-seq library preparation and sequencing

ChIP DNA library construction was performed by using ThruPlex DNA-seq Kit (Rubicon Genomics, Ann Arbor, MI) according to the manufacturer’s instructions. Briefly, 10 μl of ChIP DNA or 10 ng of input DNA was used for library construction and PCR amplification was performed to obtain the final library. AMPure XP beads were used for library purification and purified libraries were analyzed in Bioanalyzer 2100 (Agilent Technologies, Santa Clara, CA) to verify fragment size after amplification, library size and concentration before clustering. A total of 10pM of the library was then used for single-end sequencing on the HiSeq 2500 at the Genomic and RNA Profiling Core in Baylor College of Medicine. Sequences (51-nt reads) were aligned to the mouse genome (mm9) using the BWA algorithm. Aligns were extended in silico at their 3’-ends to a length of 150bp and assigned to 32-nt bins along the genome. The resulting histograms were stored as Binary Analysis Results (BAR) files. Peak locations were determined using the Model-based Analysis of ChIP-seq Algorithm (MACS) with a *p*-value cutoff of 1E-8.

#### Validation of ERα -binding sites by ChIP-quantitative PCR

ERα ChIP was performed as described above in the ChIP-seq analysis. ChIP DNA and input DNA were analyzed by qPCR using SYBR Green Master Mix. Primers were generated corresponding to the regions identified by our ChIP-seq. The enrichment of ERα-binding found in each sample was normalized to input values. For primer sequences, see S1 Table.

#### Bioinformatics

The web-based application Galaxy (http://galaxyproject.org) was used to intersect the binding regions from the ChIP-seq replicates [34–36]. Cross-correlation analysis was performed using phantompeakqualtools (https://www.encodeproject.org/software/phantompeakqualtools/) [37]. Cistrome (http://cistrome.org/ap/) was used to construct the correlation plot, conservation plot, and SeqPos Motif discovery analyses [38]. HOMER (Hypergeometric Optimization of Motif EnRichment) was used for Motif Discovery [39]. Integrative Genomics Viewer (IGV) was used to generate custom annotation tracks to view ERα binding in relation to specific genes (http://www.broadinstitute.org/igv). Enriched gene ontology (GO) terms were identified using the DAVID Functional Annotation Tool (http://david.abcc.ncifcrf.gov/summary.jsp) [40]. Principle component analysis and Euclidean distances-based clustering were performed using the normalized and log-transformed read counts.

#### Accession numbers

RNA-seq and ERα ChIP-seq data were deposited in the Gene Expression Omnibus (GEO, Accession number GSE130032).

#### Statistical analysis

Data are presented as mean±SEM. The significance of the differences between groups was determined by Student's *t*-test. Values were considered statistically significant at *p* < 0.05.

## Results

### Genome-wide analysis of ERα binding sites

**Fig 1:**
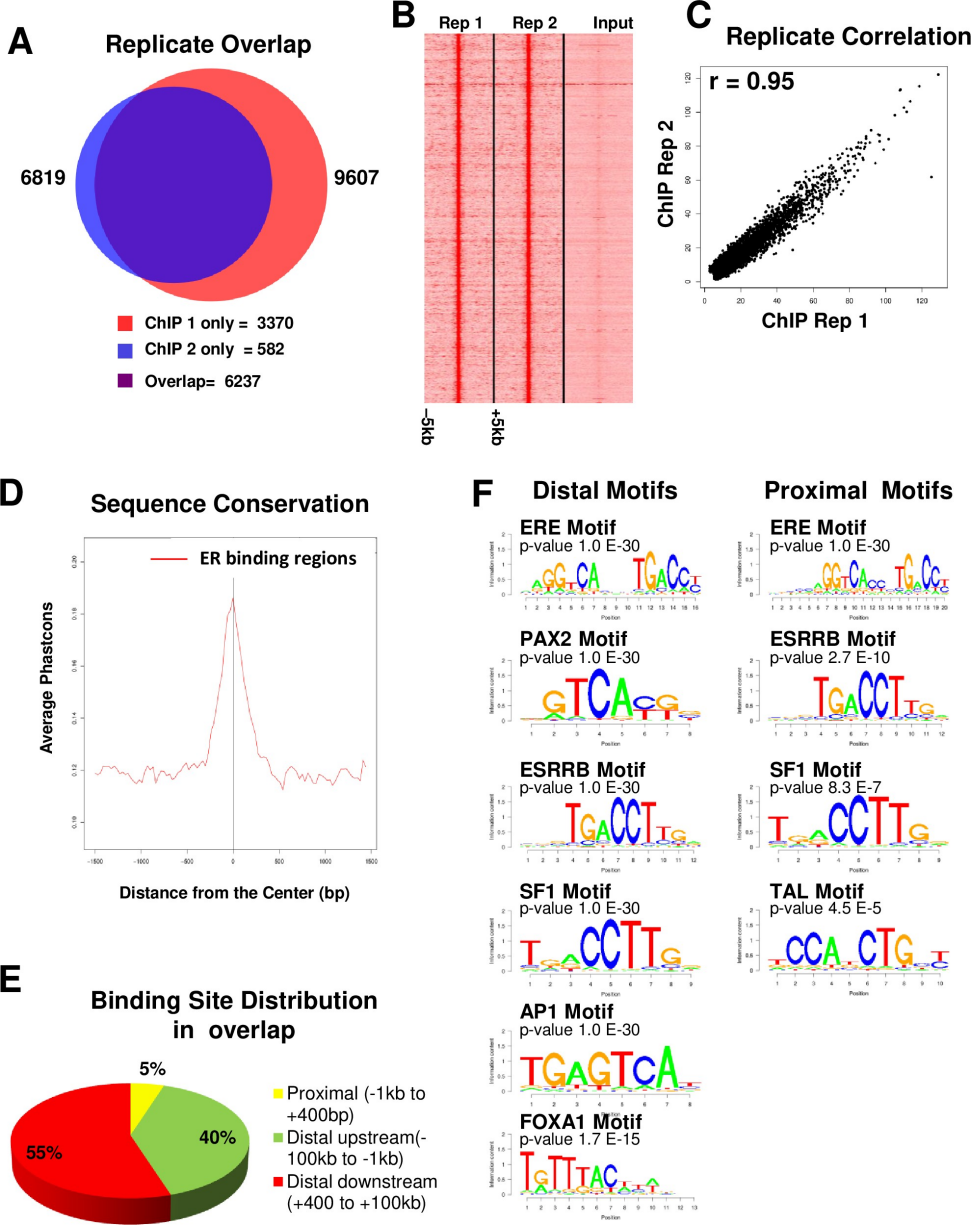
Validation of ERα ChIP-seq accuracy, binding sites distribution and motif enrichment. (A) Proportional Venn diagram representing the intersection of ERα binding sites identified in two ChIP-seq replicates; Six mice per replicate. (B) Heatmap showing ERα binding events found in the mammary gland after 2h of treatment with estradiol (ERα ChIP-seq replicate 1 and 2 [left] and input [right]). The window shows ±5kb regions from the center of the binding sites. (C) Pearson correlation of the ERα binding sites of two ChIP-seq replicates (r = 0.95). (D) Conservation plot of mouse ERα binding sites with high conservation around peak centers compared to flanking regions. (E) Distribution of ERα binding sites in the overlap of ERα binding events were identified in two ChIP-seq replicates. (F) SeqPos motif enrichment in distal (up and down) and proximal regions of ERα binding sites.

We mapped ERα binding events using chromatin immunoprecipitation followed by deep sequencing on mammary glands collected from ovariectomized mice that had been treated with estradiol for 2 hours. To ensure a robust and precise representation of the ERα binding sites in the mammary gland, we employed two independent biological replicates for ERα ChIP-seq analysis. Binding events were called using the MACS model based peak finding algorithm. A *p-*value cutoff 1E-8 identified 9607 binding regions in replicate 1 and 6819 binding regions in replicate 2. The total number of reads and uniquely mapped reads for each sample is shown in S2 Table. By using the web-based application Galaxy/Cistrome, intersection of the two biological replicates showed 78% of binding site concordance. This analysis yielded 6237 common ER binding events in mammary epithelial cells mapped near 3686 unique genes after acute treatment with estradiol (Fig 1A). A heatmap was generated for visualization of the 6237 common ERα peaks found in both replicates (Fig 1B). We used Pearson’s correlation method to compare two biological replicates. As shown in Fig 1C, Pearson’s correlation was very high (r = 0.95) between the two biological replicates suggesting that good reproducibility between the two replicate samples. Comparison of the sequences from ER intervals within various placental mammalian genomes showed a strong level of conservation in the regions of ERα binding only and not in surrounding regions (Fig 1D). Furthermore, we also performed cross-correlation analysis in ERα ChIP replicate 1 and 2. We observed two cross-correlation peaks one corresponding to the read length (Phantom peak) and other one to the average fragment length of library suggesting that our ChIP-seq is robust (S1 Fig).

We next analyzed ERα binding site distribution in this data set. ERα binding sites were divided into three categories; distal upstream (-100kb to -1kb), proximal (-1kb to +400 bp), and distal downstream (+400 bp to +100kb), based on the peak locations relative to RefSeq genes. In this analysis, binding sites were assigned to the nearest transcription start site (TSS). As shown in Fig 1E, only 5% of ERα binding sites were distributed in the proximal region. However, majority of ERα binding sites were located in the distal upstream (40%) and downstream (55%) regions. These results indicate that the vast majority of ERα binding sites are located at the distal regions of ER target genes in the mouse mammary gland which is consistent with ER binding sites in ER-positive breast cancer cells [21–23]. Further, it has been shown that distal ERα binding sites are anchored at gene promoters through long-range chromatin interactions, suggesting that ERα functions by bringing genes together for coordinated transcriptional regulation by chromatin looping [41]

### Motif analysis

To discover the network of transcription factors linked with genomic binding of ERα, we employed the Cistrome tool SeqPos and identified enriched binding motifs within the proximal and distal regulatory regions of ERα binding sites. In the distal regions (up and down) of ERα binding sites contained a canonical ERE and other motifs including PAX2, ESRRB, SF1, and AP1 motifs as the most highly enriched (p-value <10^−30^) cis-elements. Additionally, there was substantial enrichment of FOXA1 motif in the distal ERα binding regions which is consistent with previously reported data that FOXA1 acts as an important pioneer factor for ER chromatin interaction in luminal breast cancer cells [27–29]. The proximal region (-1kb to +400bp) of ERα binding sites contained canonical ERE as the most significantly enriched motif, along with other significantly enriched motifs such as ESRRB, SF1 and TAL (Fig 1F). The complete motifs enrichment is included in S3 Table. Furthermore, we also performed motif enrichment analysis by using HOMER in the overlapped ER binding sites (regardless of binding site distribution). As expected, most of the motifs identified by Cistrome were confirmed by HOMER. The complete HOMER motifs enrichment is also included in S4 Table.

### Validation of ERα binding by ChIP qPCR

**Fig 2:**
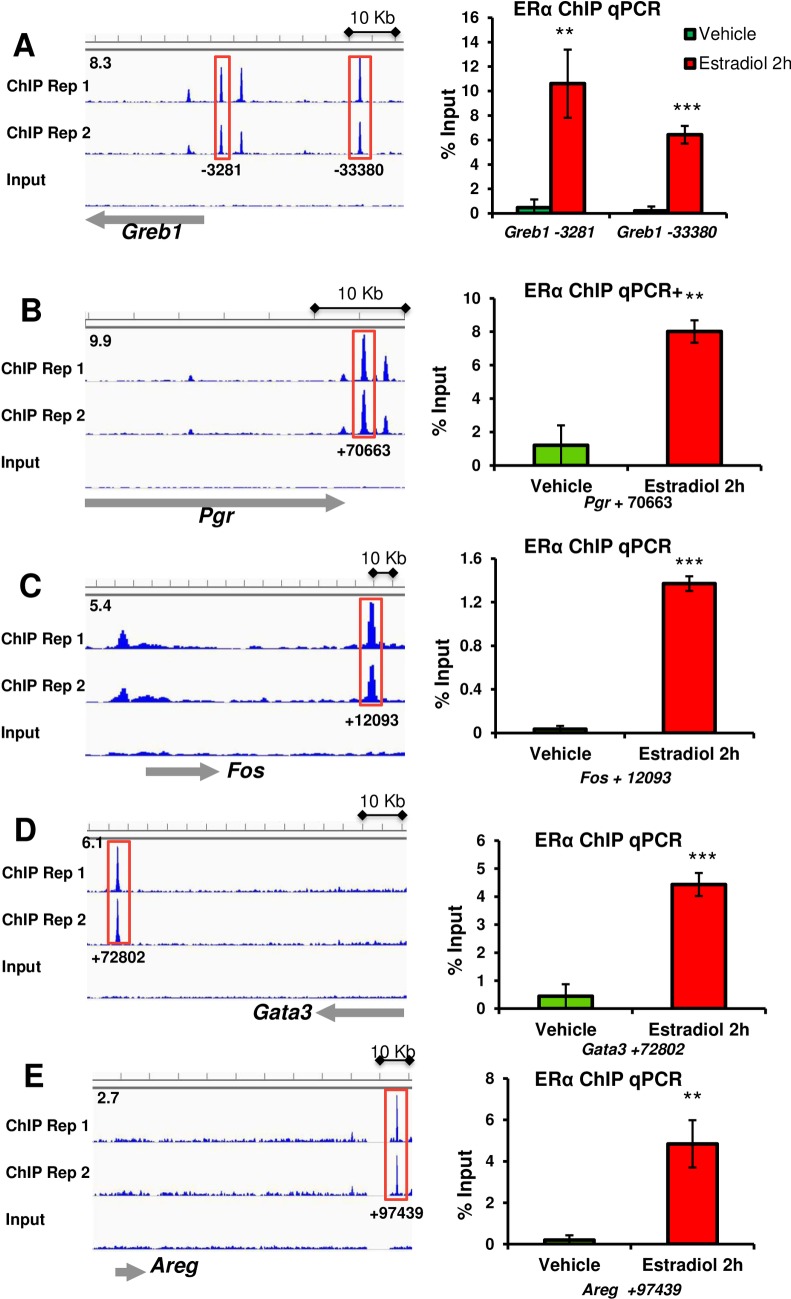
Representative screen shots of ChIP-seq data showing gene ERα recruitment at 2 hours after exposure to estradiol IGV screen shots showing ERα binding sites in relation to the TSS of (A) Greb1, (B) Pgr, (C) Fos (D) Gata3 and (E) Areg. Peak locations relative to the TSS are listed below each screen shot and the numbers indicate peak value of each gene. Red boxes represent peaks that were validated by ChIP-qPCR. Graphs representing validation of ERα occupancy using ChIP followed by qPCR for *Greb1*, *Pgr*, *Fos*, *Gata3 and Areg*. A total of six mice per replicate; Results are means ± SEM of three independent experimental replicates. **, *p* < 0.01; ***, *p* < 0.001.

We next interrogated several known estrogen-regulated genes for their ability to recruit ERα in an estrogen-dependent manner. To test this, mammary gland chromatin was isolated from ovariectomized mice that had been treated with vehicle or estradiol for 2 hours and then subjected to ERα ChIP followed by qPCR as described in the Materials and Methods. Our ChIP-seq data have identified several ERα binding sites in the distal upstream region of the *Greb1* gene (Fig 2A). Of these, we verified two of the ERα binding sites (-3281 and -33380) by ChIP qPCR. As shown in Fig 2A, ERα was highly enriched in these regions in response to estradiol treatment, confirming direct estradiol-dependent ERα recruitment to *Greb1*. Our ERα ChIP-seq analysis revealed peak of ERα binding of progesterone receptor (*Pgr*) in the distal downstream region (+70663) which was independently verified by ChIP qPCR (Fig 2B). Furthermore, our ChIP-seq data also discovered ERα binding in the distal downstream regions of *Fos*, *Gata3*, *and Areg* genes (Fig 2C–2E) and these binding sites were validated by ChIP qPCR in an estradiol-dependent manner (Fig 2C–2E). Collectively, our ERα ChIP-seq was independently confirmed by ChIP qPCR.

### Genome-wide transcriptome analysis of ER-target genes

**Fig 3:**
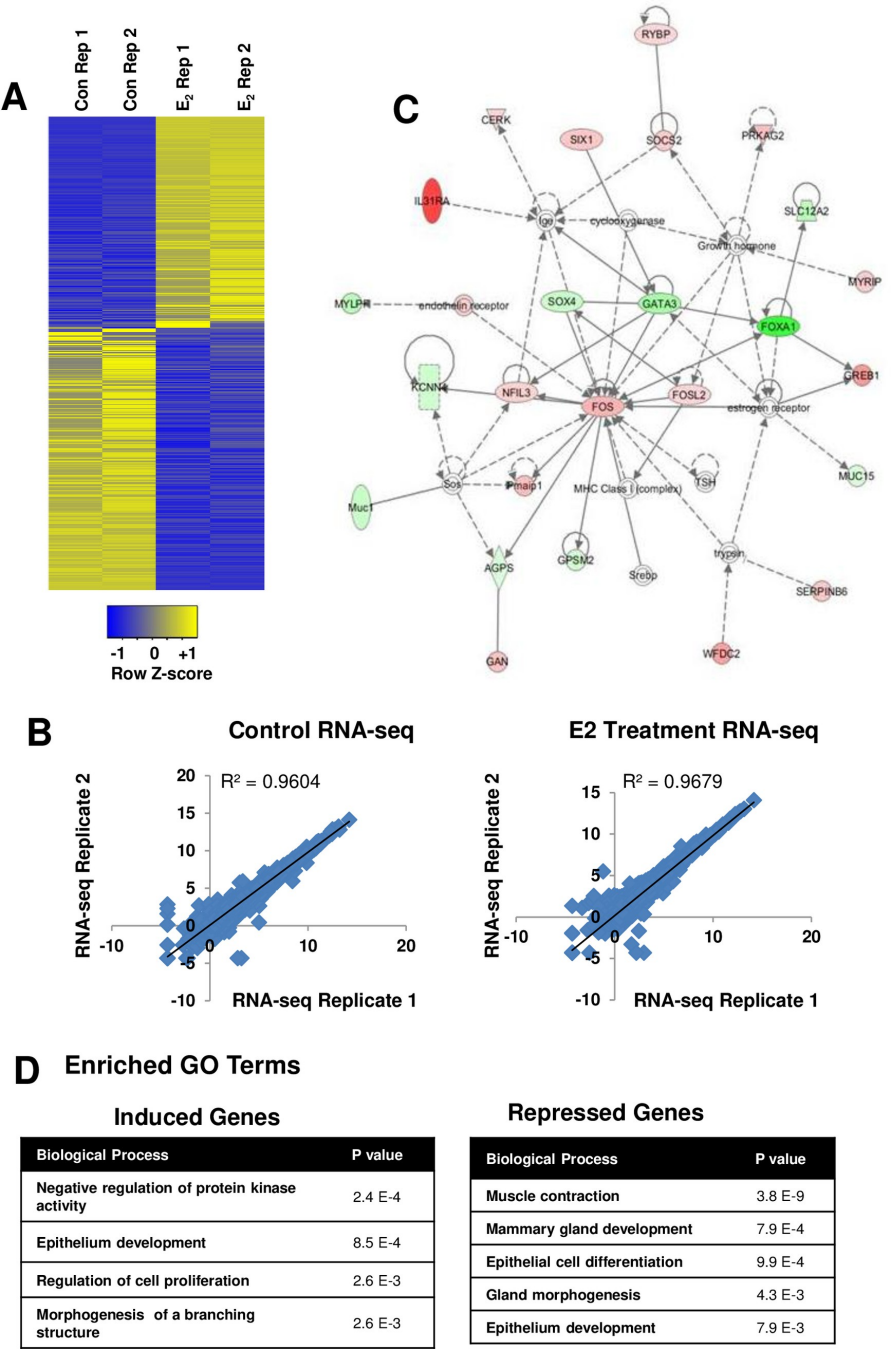
Global gene expression analysis in mouse mammary gland. (A) Heatmap of 493 genes (FDR<0.05) that are differentially expressed between mammary gland samples from vehicle (2 h) and estradiol (2 h) treatment. Duplicate pools of mammary glands from mice (5 mice per pool) were used for RNA-seq analyses under each treatment condition. (B) Pearson correlation of RNA-seq replicates (Control r = 0.96 and estrogen treatment = 0.96). (C) Ingenuity Pathway Analysis of RNA-seq. Interactions of estrogen regulated genes was analyzed by Ingenuity Pathway Analysis. A top enriched molecular network revolves around FOS. Red color indicates upregulated genes and green color indicates downregulated genes after acute treatment with estradiol. (D) Summary of enriched GO terms (FDR< 0.05) of ER target genes induced and repressed at 2 h after estradiol treatment using the DAVID Functional Annotation Tool.

To disclose the global gene signatures that are acutely regulated by estradiol, we next performed genome-wide transcriptome analysis in the mammary gland of ovariectomized mice treated with vehicle or estradiol for 2 hours. We used biological replicate pools of mammary glands from 5 mice per pool for total RNA isolation and then subjected to global gene expression analysis by RNA-seq as described in the Material and Methods. The reads were mapped to the mouse Ensembl reference set of 82,508 transcripts (annotated to 31034 genes) using TopHat. Relative transcript abundance was identified using the RSEM algorithm [42]. The edgeR package was used to identify differentially regulated gene expression [43]. The total number of reads and uniquely mapped reads for each sample is shown in S2 Table. Data quality was assessed using principal component analysis (PCA) and clustering of RNA‐seq samples using Euclidean distance. Our PCA plot of the four RNA-seq samples showed that the control and estradiol samples are well separated along the principal component 1 (PC1) which explained the 75% of the total variance (S2A Fig). Further, we also used the Euclidean sample distances to show that the samples from the same conditions are well clustered (S2B Fig). We identified 493 genes differentially regulated due to an acute treatment with estradiol. We observed that 220 genes (45%) were upregulated and 273 (55%) genes were downregulated at FDR <0.05 (S5 Table). A heatmap was also generated to show the differentially regulated genes (Fig 3A). We used Pearson’s correlation method to compare two biological replicates. As shown in Fig 3B, Pearson’s correlation was very high (r = 0.96) between the two biological replicates for both the vehicle and estradiol treated groups suggesting that our RNA-seq is robust. Next, we applied a bioinformatics approach, Ingenuity Pathways Analysis (IPA) (Ingenuity, CA), to identify a potential network based on the regulated genes to unveil the molecular mechanisms of estradiol action in the mammary gland. Using this approach, we identified β-estradiol (p = 9.46E-21) and TGFβ (p = 5.75E-19) as the top upstream regulators (data not shown). As expected, these signaling cascades were predicted to be activated by acute treatment with estradiol treatment. The most enriched gene networks were centered on FOS, a classical estrogen target gene (Fig 3C). Studies have shown that FOS has been implicated to regulate cell proliferation, differentiation, and transformation [44, 45]. Furthermore, DAVID functional annotation analysis was also used to identify the biological function of differentially regulated genes. Gene signatures-induced by estradiol were enriched for GO terms associated with negative regulation of protein kinase activity, epithelium development, regulation of cell proliferation and morphogenesis of a branching structure. However, genes repressed by estradiol were enriched for GO terms associated with muscle contraction, mammary gland development, epithelial cell differentiation, gland morphogenesis and epithelium development (Fig 3D). The complete GO terms analysis is included in S6 Table.

### Validation of RNA-seq by qPCR

**Fig 4:**
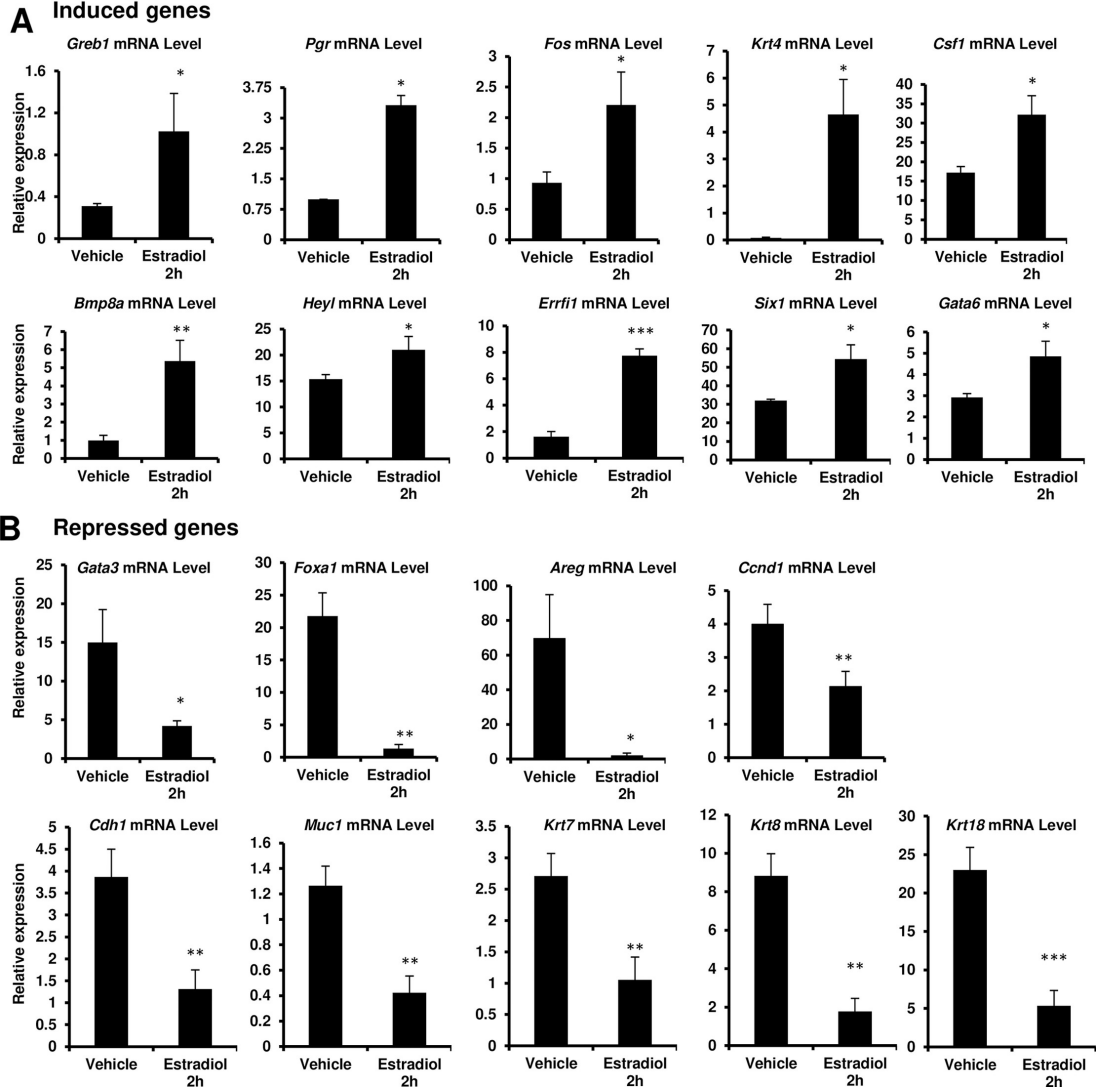
Validation of estrogen regulated genes by qPCR. Quantitative Real-Time PCR validation of estradiol-induced (A) and repressed (B) genes. Expression of selected genes was normalized using *Ppid* as the internal control. A total of 4–5 mice per treatment replicate, tested in triplicate per treatment group. Results are means ± SEM of three independent experimental replicates. *, *p* < 0.05; **, *p* < 0.01; ***, *p* < 0.001.

To validate our RNA-seq data, we measured the expression of well-established estradiol responsive genes by qPCR. To examine this, 6-week old female mice were ovariectomized and then rested for 10 days. After 10 days, mice were treated with vehicle or estradiol for 2 hours and #4 mammary glands were harvested. We used triplicate pools of mammary glands from vehicle or estradiol treated mice (4–5 mice per pool) for RNA isolation and subjected to qPCR. First, we tested estradiol-induced genes. Studies have shown that *Greb1*, *Pgr*, and *Fos* are canonical ER target genes [27–29]. Our RNA-seq analysis revealed that these genes were induced more than 2-fold by acute treatment with estradiol. Similar to what we observed in the RNA-seq data, acute estradiol treatment caused a significant increase in mRNA levels of *Greb1*, *Pgr*, and *Fos* expression in the mammary gland (Fig 4A). Our ChIP-seq analysis also identified ERα binding sites near these genes and confirmed direct hormone-dependent ER recruitment to these genes (Fig 2A–2C). Interestingly, our RNA-seq also identified several known genes (*Krt4* and *Csf1*) as well as previously unidentified estrogen target genes such as *Bmp8a*, *Heyl*, *Errfi1*, *Six1*, and *Gata6*. These estrogen-induced genes were independently confirmed by qPCR (Fig 4A). Our RNA-seq analysis also revealed that acute *in vivo* treatment with estradiol significantly downregulated *Gata3*, *Foxa1*, *Areg*, and *Ccnd1* expression. These downregulated genes were independently confirmed by qPCR. As shown in Fig 4B, acute estradiol treatment caused more than a 2-fold reduction of *Gata3*, *Foxa1*, *Areg*, and *Ccnd1* mRNA levels when compared with vehicle treatment. The possible explanation for this observation is that a secondary transcriptional response may be involved in induction of these genes in response to estradiol signaling. Our acute treatment (2 hours) makes it unlikely that we will detect secondary changes in transcription. Further, it has been shown that estradiol treatment (24h) did not increase the *Ccnd1* and *Areg* mRNA levels. However, in the presence of progesterone treatment, these genes were dramatically increased suggesting that progesterone may also be required for these gene expression in the normal mouse mammary gland [46]. Indeed, Nuclear Receptor Signaling Atlas (NURSA) transcriptomine database revealed that *Foxa*1 and *Gata3* are downregulated in response to estradiol. Studies have shown that GATA3 is necessary for luminal epithelial cell differentiation and the gene is often mutated in human breast cancer [47–54]. Several direct downstream targets of GATA3 in the luminal epithelium have been identified including FOXA1, an important regulator of ERα expression. Notably, our ERα ChIP-seq identified a peak near the *Gata3* gene and confirmed direct estrogen-dependent ERα recruitment to *Gata3*. In addition, we also observed significant downregulation of several luminal cell makers such as *Cdh1*, *Muc1*, *Krt7*, *Krt8 and KRT18* gene sets in acute treatment with estradiol treatment suggesting that maturation of luminal cells may be reduced (Fig 4B). Collectively, our data suggests that acute treatment with estradiol represses luminal cell differentiation leading to expansion of a de-differentiated epithelial cell population.

### Identification of ERα targetome in mammary epithelial cells

**Fig 5:**
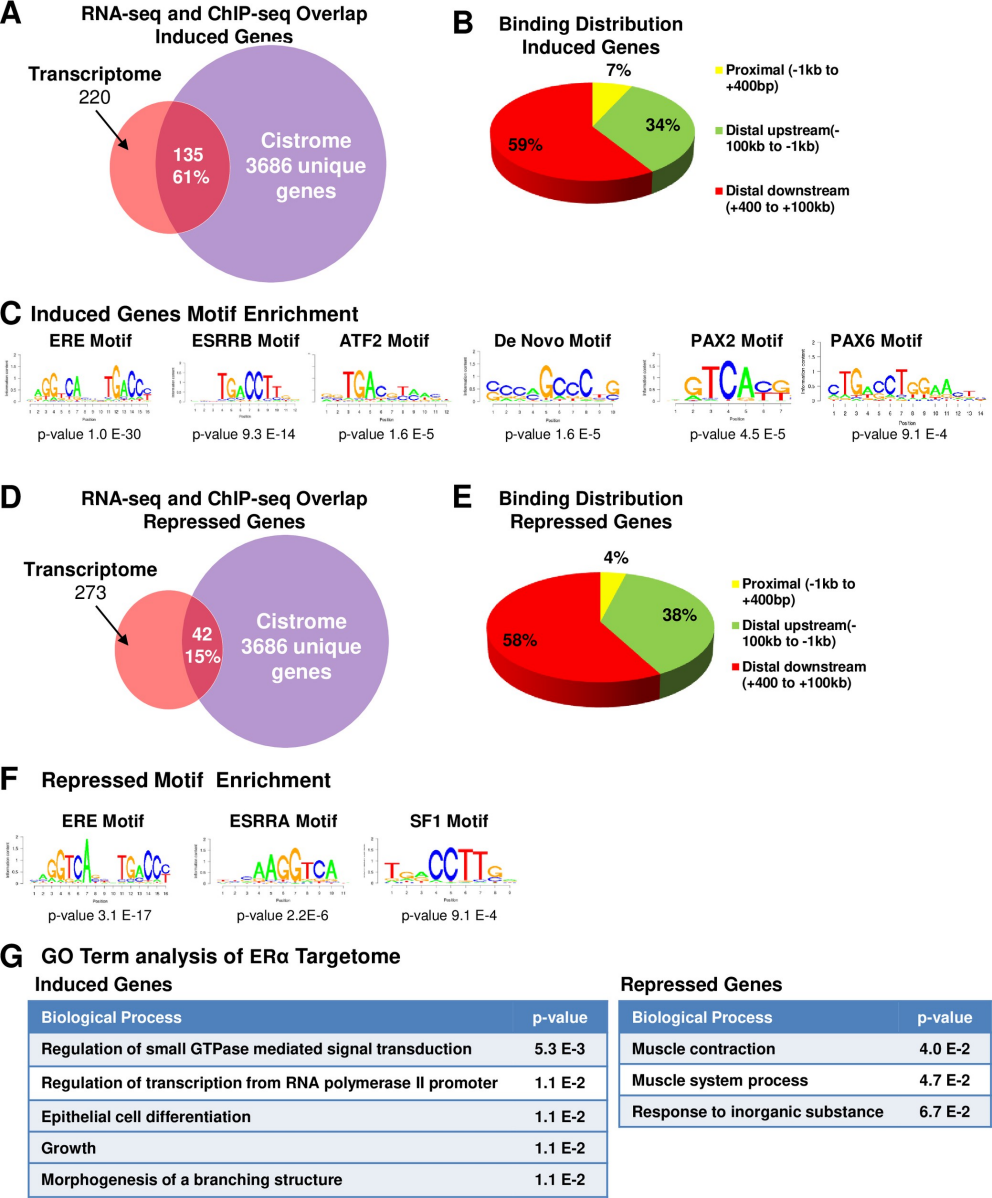
ERα targetome in mammary epithelial cells. (A) Proportional Venn diagram representing unique estradiol-induced genes at 2h has identified by RNA-seq (orange), unique genes with at least one ERα -binding region as detected by ChIP-seq (purple), and overlap indicates-induced genes at 2 h with at least one ER binding site. (B) ERα binding distribution at genes induced by estradiol at 2 h. (C) SeqPos Motif enrichment in direct ER target genes was induced at 2 h after estradiol exposure. (D) Proportional Venn diagram representing unique estradiol-repressed genes at 2 h as detected by RNA-seq (orange), unique genes with at least one ERα -binding region as detected by ChIP-seq (purple), and estradiol-repressed genes at 2 h with at least one ERα binding site (overlap). (E) ERα binding distribution at genes was repressed by estradiol treatment. (F) SeqPos Motif enrichment in the direct ER target genes repressed at 2 h after estradiol exposure. (G) Enriched GO terms of ER targetome by using the DAVID functional annotation tool.

To identify subsets of estrogen-regulated genes that are direct targets of ER positive luminal epithelial cells, we integrated ERα ChIP-seq and RNA-seq data to uncover the subset of estrogen regulated genes that directly recruit ERα in ER positive luminal epithelial cells. In this analysis, we used the 220 estradiol-induced and 273 repressed genes identified after acute treatment with estradiol (Fig 3) to interrogate the 3686 unique ER binding genes. This integration showed that 36% (177 genes) of the 493 differentially regulated genes (220 induced and 273 repressed genes) directly recruit ERα (Fig 5A and 5D). These genes (177) are shown in S7 Table. Since ERα is expressed only in a subset of luminal epithelial cells, these genes could potentially represent a global estradiol-regulated luminal epithelial cell targetome of ERα.

We next analyzed ERα binding site distribution in the ERα targetome in mammary epithelial cells. Interrogation of the ERα binding site distribution in upregulated genes showed that only 7% ERα binding sites were distributed in the proximal region, defined as -1 kb to +400 bp. However, the vast majority of ERα binding sites were located in the distal upstream (defined as -1kb to -100kb) and downstream regions (defined as +400 bp to +100kb), 34% and 59%, respectively (Fig 5B). We performed the same analysis on genes repressed by estradiol treatment. As shown in Fig 5E, only 4% of genes are associated with ERα binding in the proximal region, 38% in the distal upstream, and 58% in the distal downstream. Taken together, our ERα targetome revealed that the majority of ERα binding is located in distal regions of genes in the mammary gland, which is consistent with previous reports of ER binding sites in ER-positive breast cancer cells [21–23].

To identify the network of transcription factors linked with the ERα targetome, we employed the Cistrome tool SeqPos and identified enriched binding motifs within the estradiol-induced and repressed genes associated with ER binding regions. ER target genes induced by estradiol contained a canonical ERE motif as the most highly enriched cis-element. Additionally, ESRRB, ATF2, de novo, PAX2, and PAX6 motifs were enriched in estradiol-induced genes (Fig 5C). However, ERα binding sites in genes repressed by estradiol showed a significant enrichment of ERE, ESRRA, and SF1 motifs (Fig 5F). The co-enrichment of PAX2 sites with ERα binding is consistent with a recognized role for PAX2 in human breast cancer cells [55]. PAX2 was shown to be expressed in a subset of breast cancers and was recruited to the ER binding site after both estrogen and tamoxifen treatment [55].

To identify the biological function of the ERα targetome, we again used DAVID functional annotation analysis. Interrogation of the 135 estrogen-induced ERα targetome identified a significant enrichment for GO terms associated with regulation of small GTPase mediated signal transduction, regulation of specific transcription from RNA polymerase II promoter, epithelial cell differentiation, growth and morphogenesis of a branching structure. On the other hand, analysis of the 42 genes comprising the estrogen-repressed ER targetome showed enrichment for GO terms associated with muscle contraction, response to inorganic substance, and response to inorganic substance (Fig 5G). The complete GO terms analysis is included in S8 Table.

## Discussion

In this study, we report for the first time a comprehensive analysis of ERα binding events which elucidates the molecular mechanism of ERα action in the normal mouse mammary gland *in vivo* in response to acute estradiol treatment. We have identified 6237 high-confidence ERα binding sites in two biological replicates. These binding regions also corresponded with 3686 unique genes in the mouse genome that recruit ERα in response to acute estradiol treatment. Recent studies have shown that ER interacts with DNA sites in the absence of ligand in breast cancer cells and mouse uterus [56, 57]. However, these ER chromatin binding sites are relatively lower than ligand–induced ERα binding sites. It has also been shown that ER binds the same chromatin binding sites regardless of the ligand; however, signal intensity was highest for estradiol treatment [29]. The distribution of ERα binding sites revealed that the majority of these sites (95%) are located in the distal enhancer, which is in agreement with other studies in ER-positive breast cancer cell lines [21, 22]. Our data also suggest that ERα interactions occur at distal sites under normal physiological conditions. Interrogation of ERα-DNA interaction sites revealed ERE, PAX2, SF1, and AP1 motifs were highly enriched at sites where ERα binds, suggesting that these transcription factors interact with DNA and contribute to stabilizing the ER complex on chromatin. Furthermore, our ER binding sites also identified the FOXA1 motif, whose expression is required for luminal epithelial ERα expression and post-pubertal development of the normal mammary gland [58]. FOXA1 is an established pioneer factor for the majority of ERα binding to the genome in human breast cancer cells [21, 29]. Whole transcriptome analysis by RNA-seq revealed that 493 genes are differentially regulated by acute treatment with estradiol in the mouse mammary gland *in vivo*. By integrating genome-wide ERα binding and global gene expression signatures, we uncovered a novel ER targetome of 177 genes that are associated with estrogen-dependent ductal elongation in the mouse mammary gland *in vivo*. This cistromic and transcriptome resource revealed the cis-regulatory elements and cofactors that are involved in estrogen signaling in the normal mouse mammary gland.

It is well documented that the ovarian hormones estrogen and progesterone act as master regulators of mammary gland development. Specifically, estrogen triggers ductal elongation during puberty whereas progesterone is involved in mammary gland side-branching [46, 59–61]. Studies have shown that estradiol-mediated activation of ERα signaling is required for ductal mammary epithelial cells to proliferate, leading to ductal outgrowth. Deletion of ERα results in normal mammary glands before puberty. However, after puberty, terminal end buds remained absent, and the failure of ducts to invade into the fat pad, suggesting that ERα action is essential for mammary gland development [16, 62]. Only subset of luminal epithelial cells are ERα positive, and release of paracrine signals from these ER-expressing cells permit other nearby epithelial cells, both luminal and myoepithelial, to participate directly in ductal outgrowth [16, 18].

In our whole genome transcriptome profiling using RNA-seq, we identified that ER-regulated genes were affected by acute treatment with estradiol in the mouse mammary gland *in vivo*. We discovered that 493 genes are differentially regulated by estradiol. According to DAVID functional annotation analysis, these differentially regulated genes are primarily involved in molecular and cellular processes ranging from epithelium development to morphogenesis of a branching structure. Furthermore, we validated the RNA-seq results independently by qPCR analysis to confirm that these genes were indeed regulated in the mammary gland in response to acute treatment with estradiol. For validation, we selected canonical ER target genes as well as previously unidentified ER target genes. Studies have shown that *Greb1*, *Pgr*, and *Fos* are classical estradiol target genes and are strongly induced by estradiol. ERα directly controls GREB1 expression, and GREB1 is required for breast cancer cell growth. Clinically, the loss or reduced expression of GREB1 is predictive of poor outcome and decreased relapse free survival, similar to ER status [63–67]. It is well documented that PR is an ER target gene and induces proliferation of neighboring cells primarily by a paracrine mechanism [68]. It has been shown that progesterone induces receptor activator for nuclear factor κB ligand (RANKL) secretion from ERα+/PR+ mammary cells, and then this factor can directly binds to its receptor RANK to induce side-branching and alveolar development during ductal development and pregnancy [59, 69].

Fos is an immediate early gene acutely induced by intracellular signaling cascades [70]. Fos, along with other members of the Fos family, dimerize with Jun to form the AP-1 transcription factor complex which regulates many genes involved in proliferation, differentiation, and survival. In breast cancer cells the Fos gene also plays a key role in tumorigenesis and invasive growth. Our data showed that estradiol induces expression of *Greb1*, *Pgr*, and *Fos* genes in the normal mouse mammary gland. Furthermore, our ERα ChIP-seq data confirmed that ER binds these genes at the distal region.

BMP8a is a member of transforming growth factor β (TGFβ) and plays a central role in cell proliferation, differentiation, and survival [71]. A recent study showed that BMP8a acts as paracrine factor for estrogen-dependent regulation of epithelial cell proliferation in the uterus [72]. In the present study, we observed acute exposure to estradiol induced *Bmp8a* mRNA levels in the mammary gland, suggesting that *Bmp8a* may also act as a paracrine effector of estrogen-induced mammary epithelial cell proliferation. Furthermore, our ERα ChIP-seq data also identified ERα binding near *Bmp8a* gene, which was independently confirmed by ChIP qPCR (data not shown), suggesting that *Bmp8a* is also a direct target of ERα in the normal mammary gland.

GATA3 has been implicated in breast tumorigenesis and its highest expression was observed in the luminal subtype of breast cancer [73, 74]. Mutations in GATA3 have also been identified in a subset of breast cancers [75]. In the normal mouse mammary gland, *Gata3* is the most highly expressed transcription factor in the mammary epithelium [76]. Using a mammary epithelium-specific knockout of *Gata3*, it has been shown that *Gata3* is essential in maintaining luminal epithelial cell differentiation [48, 49]. Several direct downstream targets of GATA3 in the luminal epithelium have been identified including FOXA1, a key regulator of ER binding in the breast cancer cells. Furthermore, ER binds near the GATA3 gene in breast cancer cells, as shown through ChIP assays [21, 23, 77].

Our ERα ChIP-seq data also revealed that ER binds near the *Gata3* gene in a distal enhancer, also confirmed by ChIP qPCR, suggesting that *Gata3* is a target of ER in the normal mammary gland. Furthermore, we showed that *Gata3* is a direct target for ERα through down regulation after acute estradiol treatment. Our results suggest that acute treatment with estradiol represses *Gata3* gene expression leading to expansion of a de-differentiated epithelial cell population in the mammary duct. Our study also identified that estradiol-induced down regulation of *Gata3* was associated with repression of its target genes such as *Foxa1* and *Ccnd1*. It has been also shown that GATA3 is required for CCND1expression [78]. In addition, our data suggest that *Foxa1* is involved with ERα binding in the normal mouse mammary gland, since motif analysis of ERα binding regions in the normal mammary gland showed *Foxa1* motif enrichment. Studies have shown that FOXA1 acts as a pioneer factor for ER binding in the distal enhancer sites in breast cancer cells [26, 29]. Our findings also support the notion that FOXA1 is not only involved in ER binding of breast cancer cells but also involved in ER binding in the normal mouse mammary gland. Studies have shown that the majority of ERα binding sites are located in the distal enhancer sites in breast cancer cells, similar to our results from the normal mammary gland. Indeed, it has also been shown that distal enhancer sites are known to interact with transcriptional complexes located near gene through DNA looping [41, 79]. Thus it is possible that ER binds distal enhancer regions and induces its target gene transcription through chromatin looping.

In conclusion, our study offers identification of ERα binding events across the genome under normal physiological conditions in the developing mouse mammary gland and provides a useful dataset to allow further study of estrogen signaling through its receptor. Furthermore, our findings identify cis-regulatory factors that cooperate in ERα-mediated control of gene expression in the normal mammary gland.

## Supporting information

S1 Fig Cross-correlation quality assessment of ERChIP-seq. The blue dotted line indicates the location of the phantom peak (read length) and the red dotted lines show the library fragment length. NSC-Normalized strand cross-correlation, RSC-Relative strand cross-correlation.
(PPTX)

S2 Fig Data quality evaluation of mRNA-seq. (A) Principal component analysis showing a separation between control and E2 treatment. (B) Clustering of RNA‐seq samples using Euclidean distance on normalized and log‐transformed read counts.
(PPTX)

S1 Table List of primers used in ChIP-qPCR. (XLSX)

S2 Table Details of ERα ChIP-seq and RNA-seq which includes read length, total number of reads and uniquely mapped reads. (XLSX)

S3 Table List of proximal and distal motifs in ERα ChIP-seq. (XLSX)

S4 Table Motif enrichment analysis by HOMER. (XLSX)

S5 Table Differentially regulated genes by E2 treatment. (XLSX)

S6 Table Gene ontology terms for genes induced and repressed after 2 hours of estradiol treatment. (XLSX)

S7 Table List of direct target genes. (XLSX)

S8 Table Gene ontology terms for targetome. (XLSX)
