# Supplementary figures and images for: The genomic landscape of estrogen receptor α binding sites in mouse mammary gland
Source: PLoS One. 2019 Aug 13;14(8):e0220311. doi: 10.1371/journal.pone.0220311 (PMC6692022; doi:10.1371/journal.pone.0220311)

## Slide 1
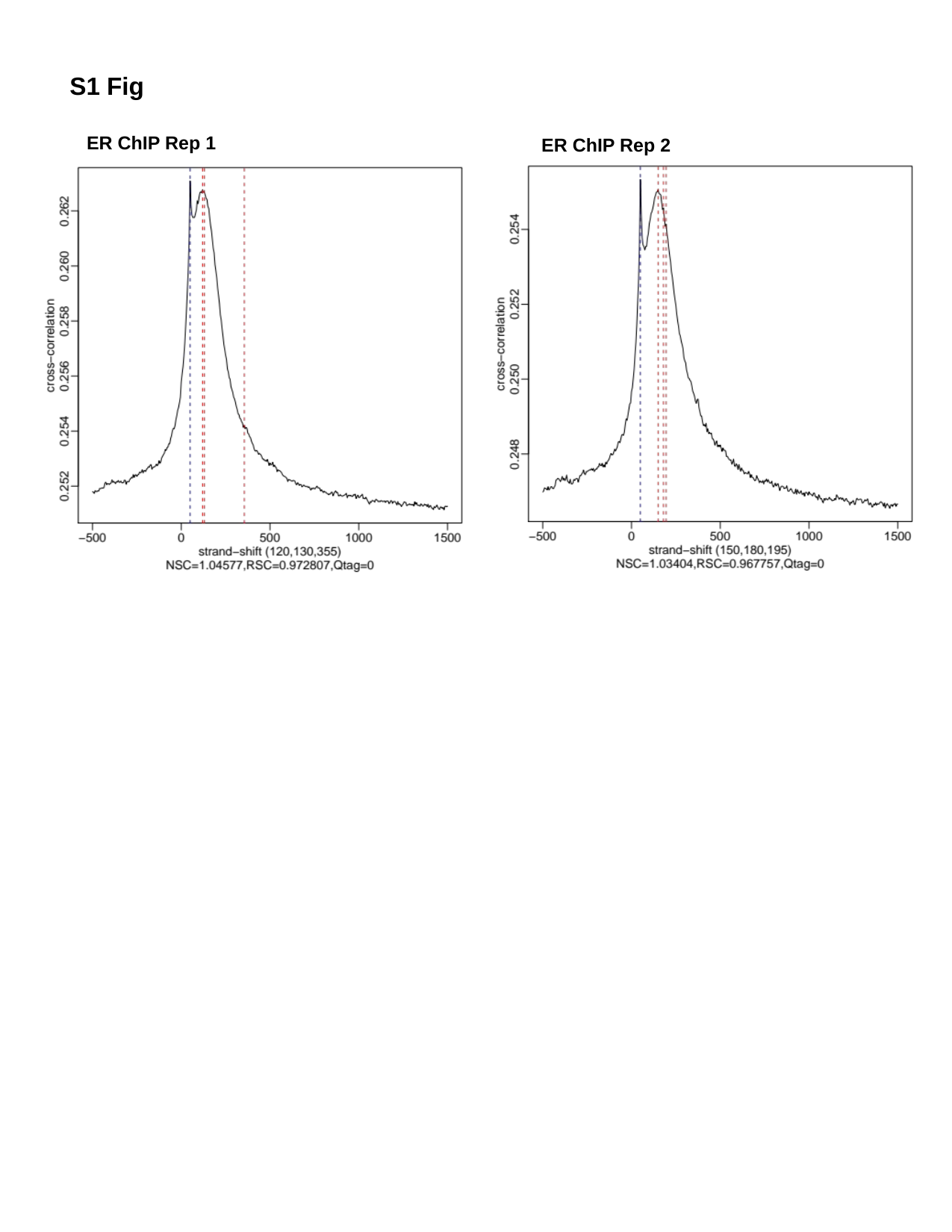

S1 Fig
ER ChIP Rep 1
ER ChIP Rep 2

Supplement: S1 Fig — The blue dotted line indicates the location of the phantom peak (read length) and the red dotted lines show the library fragment length. NSC-Normalized strand cross-correlation, RSC-Relative strand cross-correlation. (PPTX) [file pone.0220311.s001.pptx]

## Slide 1
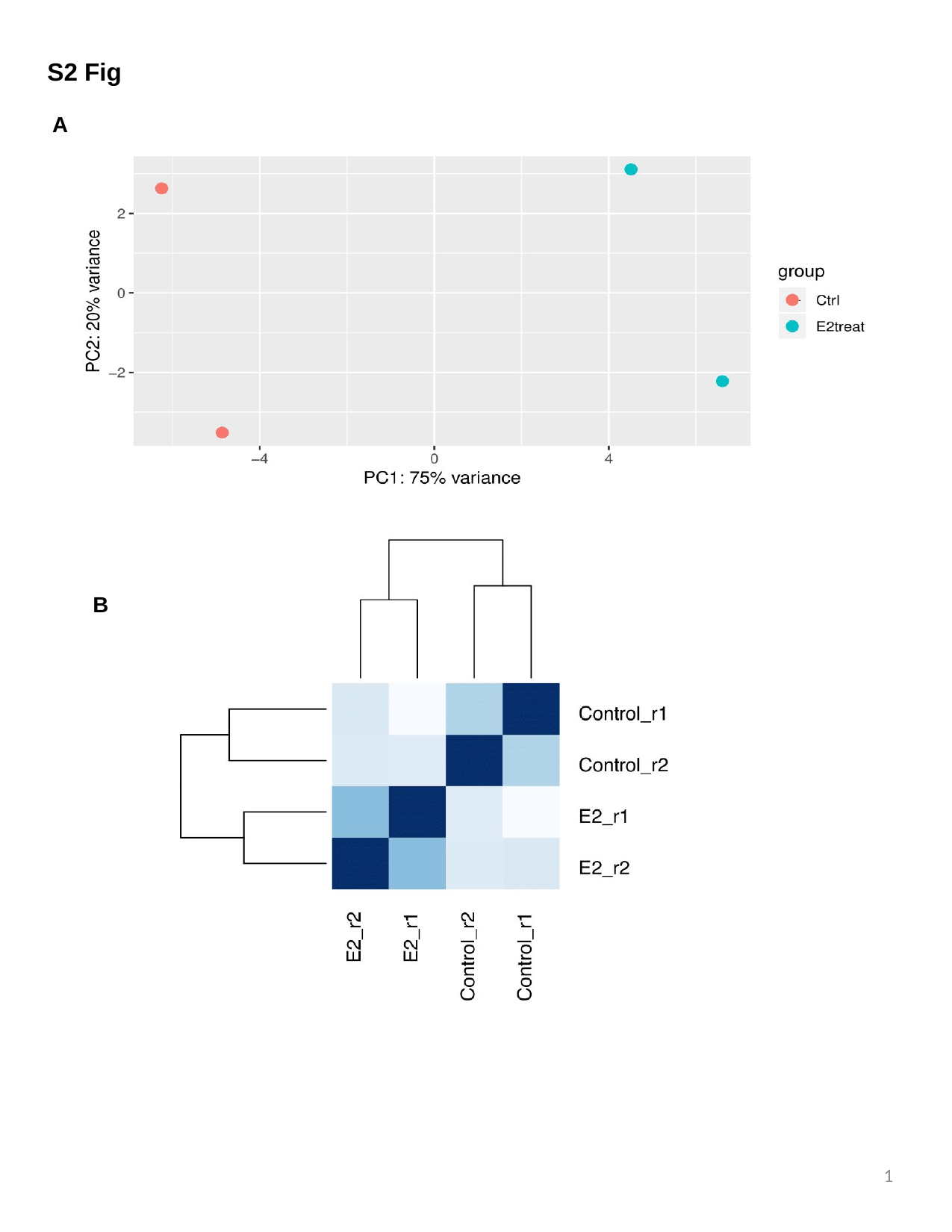

S2 Fig
A
B
1

Supplement: S2 Fig — (A) Principal component analysis showing a separation between control and E2 treatment. (B) Clustering of RNA‐seq samples using Euclidean distance on normalized and log‐transformed read counts. (PPTX) [file pone.0220311.s002.pptx]
